# Assessment of macronutrients consumption in the diet of adolescent school children in four seasons: a longitudinal study from an urban city in Pakistan

**DOI:** 10.1186/s41043-021-00268-5

**Published:** 2021-10-16

**Authors:** Syed Hasan Raza Abidi, Aysha Almas, Abdul Ghani, Sania Sabir, Romania Iqbal

**Affiliations:** 1 Aga Khan Medical College, Karachi, Pakistan; 2 Department of Medicine, Aga Khan University, Stadium Road, Karachi, Pakistan; 3 Health Department, Government of Baluchistan, Chaghi, Pakistan; 4 Department of Community Health Sciences, Aga Khan University, Karachi, Pakistan

**Keywords:** Adolescent, 24-h diet recall, Pakistan, Nutrition

## Abstract

**Background:**

A healthy diet in the adolescence period is essential for physical, mental, and immunological development. We aimed to assess macronutrient consumption in the diet of adolescent school children using 24 h recalls in four seasons of the year.

**Method:**

This was a longitudinal study conducted from February 2014 to June 2015. The study population included 155 school children aged 7–14 years from an urban school in Karachi. 24HR recall was conducted on 4 random days of the 4 main seasons. A food composition table was developed where the weight, calories, carbohydrate, fat, and protein content of the food items were listed. Macronutrients quantification was calculated by using proportional weight from the food composition table. Food groups were also assigned to each food item including vegetables, fruits, grains, protein foods, dairy products, and oils.

**Results:**

A total of 155 adolescent children aged between 7 and 14 years were approached. Out of the 155 preadolescents and adolescents, 150 (96.7%) agreed to participate. The mean (SD) age of the children was 11.31 (1.6) years, and 59% of all the children were males. Overall mean (SD) daily intake for all seasons was 195.31 (86.87) grams of carbohydrates, 94.77 (71.87) grams of proteins, and 55.87 (30.79) grams of fats. Carbohydrates formed 48.16%, protein 21.92%, and fat 29.93% of the total caloric intake. The mean (SD) daily caloric intake was 1517 (644) grams. Overall, the highest source of calories was from carbohydrate 781 (347) Kilocalories (Kcal), followed by fat 502 (277) Kcal and protein 379 (287). The Carbohydrate intake in 24 h was highest in the autumn; 212.81 (85.37), and there was a significant difference in carbohydrate intake in all seasons (*p* value 0.003). Consumption of discretionary food group was high (31.3%), and consumption of fruits and vegetables was low (29%).

**Conclusion:**

The study reports a suboptimal caloric intake of fewer than 2000 cal/day among the adolescents from school. The highest source of calories was from carbohydrates.The highest consumption of food was in autumn and the least in summer. Fruits and vegetable intake was low, and discretionary food intake was high.

## Introduction

A healthy diet in the adolescence period (10–19 years) is essential for physical, mental, and immunological development [1]. According to the World Health Organization, a deficiency, excess, or imbalance of energy and nutrients can lead to malnutrition [2]. As adolescence is a period of growth, the age group is highly susceptible to developing of malnutrition. In lower middle -income countries like Pakistan, adolescents in all provinces have a high prevalence of both underweight and overweight [3]. It is essential to assess the dietary intake of these children for monitoring dietary patterns and designing interventions.

According to the National Nutritional survey of Pakistan, 2018, 11–21% of adolescents were underweight, 10–11% were overweight, and 5–7% were obese [3]. However, the macronutrient composition of the food was not described in the survey. Accurate measures for assessment of food intake are required in order to investigate the relationship between diet and health [4]. Several methods of assessment of food intake have been used in the literature. These include estimated food records, weighed food records, food frequency questionnaires (FFQ), 24-h dietary recalls, 5-day dietary recall (5DDR), and brief dietary assessment instruments [5–8]. In food records, the respondent records the foods and beverages and their consumed amount over one or more days. This however creates a considerable burden on the participant or the parent and often are completed later from memory which may lead to recall bias [8, 9]. The FFQ asks respondents to report their usual frequency of consumption of each food from a list of foods for a specific period but this requires cognitive skills (for example, ability to average consumption) that many elementary school children lack [5, 10, 11]. The 5DDR has been used in capturing the participant’s habitual eating patterns over 2 weekend days and 3 weekdays but this is resource-intensive [6]. Brief dietary assessment instruments have been used in situations that only require assessment of a specific part of the diet [12]. For the 24-h dietary recall, the respondent is asked to recall and report all the foods and beverages consumed in the preceding 24 h. The recall typically is conducted by interview, in person, or by telephone or computer [13]. The advantage of 24-h recall is that elementary school children without parental assistance can give information about their diet over 24 h [14], hence it serves as a gold standard to address two issues facing researchers studying this age group: literacy and motivation [15–17].

Limited work has been done in Pakistan for the assessment of food intake in children. Studies have reported on nutrient consumption using the methods of assessment described above [18, 19]. However, results of these studies were only onetime dietary assessments and did not consider the seasonal variation in the food intake of these children. In addition, the use of FFQ might have over or underestimated the quantity of food intake due to need of significant cognitive skills by children. We aimed to assess macronutrient consumption in the diet of adolescent school children using 24 h recalls in 4 seasons.

## Methods

### Study design, population, and setting

It was a longitudinal study conducted from February 2014 to June 2015. School children aged 7–14 years, in class 3–7, were randomly selected from a local public school present in the East district of Karachi which houses the middle to higher-income population. Ethical approval from the Ethical review committee(2488-Med-ERC-13), Aga Khan University was taken before commencement of the study. Informed consent from the childrens’ parents and assent from the children was taken. Recall 1 was during winter, recall 2 during summer, recall 3 during autumn, and recall 4 during spring. The sampling frame was the list of children aged 7–14 years admitted to this school. The list was obtained from the school administration, and a code was assigned to each child. Epi info was used to generate random numbers from the list. The children corresponding to the random numbers were then enrolled in the study. A minimum sample size of 150 children was required to estimate a proportion of 47% carbohydrate intake in summer season in a day with 95% confidence interval and absolute precision of 0.08. WHO sample size calculator, version 2.0 was used for this purpose [20].

### 24-h dietary recall

The 24HR dietary recall was conducted as an open-ended, prompted interview by a trained research dietician. The reference period for the recall was 24 h prior to the interview. The 24HR recalls were administered on any one of 5 randomly selected days, but avoiding days immediately following important religious and social festivals. To account for variations in diet, intake days were selected to represent the four main seasons of the year and weekdays as well as weekends. Interviews were conducted to take the respondent through the recall process in direct chronological order from the first food encounter of the day to the last [21]. Information about food consumption was obtained by asking about foods eaten at meals as well as snacks eaten between meals. Portion sizes of various foods and composite dishes consumed were obtained using household measures such as tablespoons, serving spoons, bowls, and cups. For shop-bought items (e. g., bread), their characteristics (e. g., the size of the loaf and thickness of the slice) were included as well as their brand names. All recalls were conducted in the subjects’ mother tongue. When a respondent was not able to identify a prepared food item by name, only the main ingredients and the type of preparation (e.g., curry or dry) were noted. An appropriate name was then assigned to the preparation by the interviewer.

### Data entry from 24-h recall

#### Data entry

Each participant was assigned a code, and each participant had multiple food item consumptions. For example, participant 2 utilized 7 food items. Each food item was numerically coded from 1 to 170 after reviewing twenty 24HR recall forms. If new food items were found in the subsequent form, they were assigned codes beyond 170. Each food item was also categorized for consistency of food; 1 = raw, 2 = fried, 3 = boiled, 4 = cooked with water + spice + oil, 5 = baked/roasted, 6 = processed food/packet). The serving size was categorized as: 1 = whole, 2 = sliced/pieced, 3 = cup, 4 = plate, 5 = bowl, 6 = table spoon, 7 = glass or 250 ml. The corresponding weight in grams (gm) of each of this serving size was: slice = 29gm, cup = 128gm, plate = 200gm, bowl = 200gm, table spoon = 14.3gm, and Glass = 250gm. For the whole item, the corresponding weight of the item was taken from the US Department of Agriculture’s (USDA) National Nutrient Database. The quantity of each serving size was also recorded. The total weight of each food item was then calculated by multiplying the serving size weight *quantity.

#### Development of food composition table

A food composition table was developed on excel where weight in gms, calories, carbohydrate, fat, and protein content of the food items was listed. The source for most of the items was the US Department of Agriculture’s (USDA) National Nutrient Database for Standard Reference release 21 (USDA, Washington, DC, USA) [22]. For those food items which were not available on USDA, additional sources including the Bangladesh Food Composition Table (first edition -2012) was used [23]. In rare cases, if items were still not found then other websites were used (recipeofhealth.com, nutritionix.com, eatthismuch.com, dalda ka dastarkhwan).

#### Calculation of Calories and macronutrients

The corresponding standard value of each food item for calories, weight, and macronutrients was exported into the SPSS from the food composition table. Macronutrients including protein, fat, carbohydrate were calculated by using proportional weight from the food composition table. For example, if a chicken item weighing 140 g had 42-g protein, the protein content of 200 g = 200 * 42/140 = 60 g protein. Similar calculations were done for all food items in the SPSS using compute function. Restructure variable was used to sum the food consumed along with its calories and macronutrients to get the sum of food eaten by a participant. Mean daily macronutrient and caloric consumption, overall percent macronutrient consumption, and percent macronutrient consumption according to the season was reported for each adolescent.

#### Categorization of food groups

Food groups were also assigned to each food item using the dietary guidelines for Americans [24]. A healthy eating pattern includes a variety of nutrient-dense foods across and within all the food groups: vegetables, fruits, grains, protein foods, dairy products, and oils. All food items were categorized into the mentioned food groups except for oils due to the type of food that is prepared in this region, and a proper categorization would be quite resource-intensive, hence this is a limitation in our study.

### Statistical analysis

Mean and standard deviation was used for quantitative variable while frequency and percentage were used for qualitative variables. Analysis of variance was used to compare the results among the 4 different recalls over the year. *P* value < 0.005 was considered as significant. Statistical package for social sciences version 20.1 was used for analysis.

## Results

A total of 155 adolescents aged between 7 and 14 years were approached. Out of the 155 preadolescents and adolescents, 150 (96.7%) agreed to participate. All participants participated in 3 recalls, however, 115 (76.6%) participated in the 4th recall. A total of 565 observations for 24-h recall was recorded over the year. The mean (SD) age of the children was 11.31 (1.6) years, and 59% of all the children were males. Table 1 shows the demographics and class distribution in the school of the participants.

**Table 1:** Demographics of the adolescent school children (*N* = 150)

	*n* (%)
Mean age (SD)	11.3 (1.6)
Age groups	
7–10 years	46 (30.7)
11–14 years	99 (66)
Gender	
Boys	88 (58.6)
Girls	61 (41.3)
Class	
III	27 (18)
IV	28 (18.7)
V	34 (22.7)
VI	29 (19.3)
VII	32 (21.4)

**Fig. 1:**
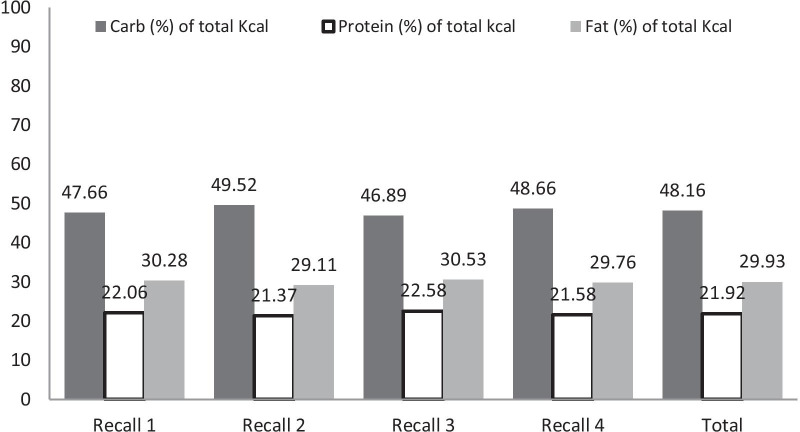
Contribution of carbohydrate, protein, and fat in total daily caloric intake

Overall mean (SD) daily intake of carbohydrates for all seasons was 195.31 (86.87) g, 94.77 (71.87) g of proteins, and 55.87 (30.79) g of fats. The Carbohydrate intake in grams in 24 h was highest in autumn; 212.81 (85.37), and there was a significant difference in carbohydrate intake in all seasons (*p* value 0.003). Similarly, the protein intake was 108.65 (80.48) g, and Fat intake was 64.62 (35.32) g which were also the highest in autumn (Table 2 shows the Mean Daily Macronutrient Consumption in Grams for School Going Children in 4 seasons). It was also observed that carbohydrates formed the major component of the children’s diets making up around 48.16% of the total caloric intake in all four seasons. Protein content was seen to be the lowest overall making only 21.92% of their diets on average. The fat content was also seen to be consistent among all the recalls averaging about 29.93% of the children’s diets. Figure 1 shows the percentage of macronutrient component eaten by an adolescent in 24 h in the 4 recalls.

**Table 2:** Mean Daily Macronutrient Consumption in Grams for school going adolescents in the 4 seasons of a year (*N* = 150)

	Average of 4 recalls	Recall 1 (winter)	Recall 2 (summer)	Recall 3 (autumn)	Recall 4 (spring)*	*p* value**
	Mean (SD)	Mean (SD)	Mean (SD)	Mean (SD)	Mean (SD)	
Carbohydrate (g)	195.31 (86.87)	200.99 (91.59)	177.02 (84.39)	212.81 (85.37)	189.08 (81.32)	0.003
Protein (g)	94.77 (71.87)	98.93 (75.95)	78.9 (52.47)	108.65 (80.48)	92.1 (73.17)	0.003
Fat (g)	55.87 (30.79)	59.99(35.38)	46.11 (21.11)	64.62 (35.32)	51.89 (24.08)	< 0.001

*n = 115, as 35 participants had lost to follow-up, mainly had left school

**p value is between the 4 recalls

The mean (SD) daily caloric intake was 1517 (644) g. Overall, the highest source of calories was from Carbohydrate 781 (347) Kcal, followed by fat 502 (277) Kcal and protein 379 (287) Kcal. The percent energy derived from these macronutrients was 47% from carbohydrates, 23% from protein, and 30% from fat. The Carbohydrate caloric intake in 24 h was highest in the autumn; 851.25 (341.46) Kcal, and there was a significant difference in carbohydrate intake in all seasons (*p* value 0.003). Similarly, the protein 434.59 (321.91) Kcal and Fat intake 581.62 (317.86) Kcal was also the highest in autumn. Table 3 shows the Mean Daily Caloric Intake from Macronutrients of School Going Children in 4 seasons.

**Table 3:** Mean daily caloric intake from macronutrients of school going adolescents in the 4 seasons of a year (*N* = 150)

	Average of 4 recalls	Recall 1 (winter)	Recall 2 (summer)	Recall 3 (autumn)	Recall 4 (spring)*	*F* statistic value	*p* value**
		Mean (SD)	Mean (SD)	Mean (SD)	Mean (SD)		
Calories from carbohydrate (Kcal)	781 (347)	803.97 (366.36)	708.07 (337.57)	851.25 (341.46)	756.34 (325.28)	4.766	0.003
Calories from protein (Kcal)	379 (287)	395.71 (303.82)	315.6 (209.9)	434.59 (321.91)	368.4 (292.68)	4.627	0.003
Calories from fat (Kcal)	502 (277)	539.87 (318.46)	415 (189.95)	581.62 (317.86)	466.99 (216.7)	11.212	< 0.001
Overall total mean caloric intake (Kcal)	1517 (644)	1590 (720)	1327 (521)	1698 (691)	1454 (541)	9.967	< 0.001

*n = 115, as 35 participants had lost to follow-up, mainly had left school

**p value is between the 4 recalls

**Fig. 2:**
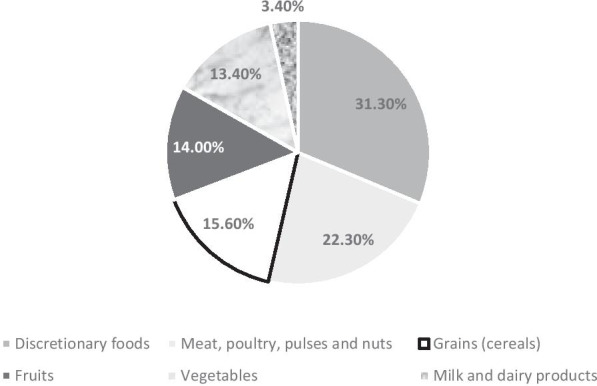
Food groups of the diet consumed by children overall

We also stratified the 24-h dietary intake according to various food groups which reported that most of the food items consumed during the four seasons belonged to the discretionary food group (31.3%) while the food group least consumed was milk and dairy products (3.4%). Other food groups consumed included meat, poultry, pulses, and nuts (22.3%), grains (15.6%), fruits (14.0%), and vegetables (13.4%). (Fig. 2. demonstrates the various food groups).

## Discussion

The dietary assessment reported carbohydrates as the major component of diet in adolescents followed by proteins and fat; a pattern similar across all 4 seasons in this study. The Caloric consumption follows a similar fashion. More diet is taken in the month of autumn compared to other seasons. Discretionary and meat group constitutes more than half of the food intake of these adolescents while fruits and vegetables constitute around one-fourth of the 24 h intake.

The adolescent period holds great importance as it is marked by a rapid physical and cognitive growth which depends upon satisfactory nutrition. Yannakoulia et al. reported a significantly higher energy intake (82 ± 31 kcal) in spring/summer season vs the autumn/winter season in 3–18-year-old Greek children, however, there was no significant difference when the adolescent group was compared exclusively [25]. Arsenault, Joanne et al. and Mitchikpe et al. also reported no significant seasonality in energy or nutrient intake [26, 27]. In Contrast, Huong, Le Thi et al. reported a significant seasonal variation in total energy intake (*p* < 0.01) with highest in autumn (1259.3 kcal) and lowest in summer (996.9 kcal) among Northern Vietnamese children. The intake of carbohydrates, protein, and lipids among children was also highest in autumn as compared to other seasons (*p* < 0.05) [28]. Several other studies exist offering reports on seasonal caloric and nutrient consumption with inconsistent results [29–34]. However, it should be noted that most of these studies were not carried out exclusively on the adolescent population. Furthermore, they compared the nutritional status only between two seasons: winter vs summer or pre-harvest vs post-harvest. This may explain the differences in our study as we reported that the highest caloric and macronutrient intake was noted in autumn while the least was noted in the summer out of the four seasons with significant seasonality noted in macronutrient consumption. Other factors which may influence nutrient intake could be several; changes in the level of activity during various seasons, vacations and even access to nutritious food at home or school depending on the socioeconomic status of the family, all which may have played a significant role in determining the results of our study [35, 36].

The adolescent period is marked by rapid growth. While females attain their adult height during this time, males continue to gain height and muscle mass [37]. FAO suggests that a recommended daily allowance of caloric intake should be 2326 kcal for girls and 2824 kcal for boys. The average total caloric intake in this study fell well below the recommended caloric intake of children of the same age. Studies reporting caloric intake among school-going adolescents are very limited, most likely as caloric intake is a complex method for reporting under-nutrition among adolescents, however, other indices, such as Body Mass Index (BMI), have widely been used [38–40]. The findings of caloric intake in our results were consistent with other studies carried out in various developing countries facing a similar crisis of malnutrition. A study carried out in India reported that the mean intake of calories varied from 1512 ± 532 kcal for pre-adolescent to 1742 ± 660 kcal for post-adolescence [41]. A study carried out in Punjab, Pakistan among adolescent girls showed that around 70% of the adolescent females were taking less than half the daily recommended intake of calories [42]. The reason for this suboptimal caloric intake in preadolescents and adolescents could be the suboptimal availability of food at home due to high cost in a middle to higher income earning neighborhood [43]. Secondly, higher use of discretionary food and less of healthy food might also be responsible for inadequate caloric intake [44].

The acceptable macronutrient distribution range (AMDR) for carbohydrates, proteins, and fats is 45–65%, 10–30%, and 25–35%, respectively, among adolescents [45]. Our study, though reports a decreased mean caloric intake, reported that the proportional intake of carbohydrates, proteins, and fats was 48.16%, 28.12%, and 29.93%, respectively, which was within the acceptable ranges. A cross-sectional study carried out including more than 11,000 school-going children in Pakistan found a low intake of proteins and fats but a high intake of carbohydrates as compared to the guidelines [46]. Studies report that being underweight or overweight is linked to socioeconomic status reflected by public sector schools or schools for the children of the affluent, as those in affluent schools tend to be overweight, and those in public sector schools tend to be underweight [40, 47]. Those from a lower socioeconomic class may have a higher proportion of carbohydrates in their diet as proteins from meat may be more costly. Additionally, introduction of discretionary food in this age group in recent years may have also increased carbohydrate consumption as seen worldwide.

A discretionary food is defined as food that is not necessary to fulfill the nutrient requirement and is consumed for enjoyment [44]. This has led to an increased proportion of carbohydrates, salts, and fat intake contributing to the burden of obesity. Majority of the nutrients consumed in this study were from the discretionary food groups. This was followed by meat while the least came from dairy products. There is scarce data on the pattern of consumption of discretionary foods in adolescents in Pakistan, however, studies from other countries show a similar trend. A study carried out in India on 1026 adolescents showed that around 70% of participants reported eating energy-dense snacks and 47% consumed three or more energy-dense beverages. Additionally, around 30% and 45% of the participants had no intake of vegetables and fruits, respectively [48]. Several other studies from both, developed and developing nations, have noticed a similar rise in intake of this food group [44, 49–52].

This study holds strength as it reports 24-h recalls at 4 different seasons in a year in the same participants with a follow-up of 76% at the time of the 4th recall. Our study reported significant seasonal variations of both, macronutrient and caloric intake, an aspect that lacks in most studies. A food composition table for adolescents was also developed as part of this study addressing the local and cultural components of the diet. However, our study also has limitations. Firstly, factors including maternal education, socioeconomic status, and physical activity can influence the results which were not considered. Secondly, the variation in intake in different seasons is based on a single days’ recall of intake instead of the typical 2–3-day recall. However, our results seem consistent with findings of other studies therefore we are confident in our results. It was also a single-centered study; hence the results cannot be generalized to the entire population of Karachi, however, it sheds light on the variability of macronutrient intake by season among school-going children which has not been captured by other studies in the region.

## Conclusion

The study reports a suboptimal food intake among this group of school-going adolescents, which is less than the standard daily allowance of > 2000 cal/day. It shows significant seasonality in caloric and macronutrient intake. The highest source of calories was from carbohydrates. The highest consumption of food (more energy production) was in autumn and the least in summer. The study also reported high consumption of the discretionary food group and low intake of fruits and vegetables among adolescents.

## Data Availability

The datasets generated and/or analyzed during the current study are not publicly available due to institutional policy but are available from the corresponding author, aysha.almas@aku.edu on reasonable request.

## Abbreviations

FFQ: Food frequency questionnaires
USDA: US Department of Agriculture’s
Kcal: Kilocalories
